# The Silent Epidemic of Diabetic Ketoacidosis at Diagnosis of Type 1 Diabetes in Children and Adolescents in Italy During the COVID-19 Pandemic in 2020

**DOI:** 10.3389/fendo.2022.878634

**Published:** 2022-06-17

**Authors:** Valentino Cherubini, Monica Marino, Andrea E. Scaramuzza, Valentina Tiberi, Adriana Bobbio, Maurizio Delvecchio, Elvira Piccinno, Federica Ortolani, Stefania Innaurato, Barbara Felappi, Francesco Gallo, Carlo Ripoli, Maria Rossella Ricciardi, Filomena Pascarella, Filomena A. Stamati, Felice Citriniti, Claudia Arnaldi, Sara Monti, Vanna Graziani, Fiorella De Berardinis, Cosimo Giannini, Francesco Chiarelli, Maria Zampolli, Rosaria De Marco, Giulia Patrizia Bracciolini, Caterina Grosso, Valeria De Donno, Barbara Piccini, Sonia Toni, Susanna Coccioli, Giuliana Cardinale, Marta Bassi, Nicola Minuto, Giuseppe D’Annunzio, Claudio Maffeis, Marco Marigliano, Angela Zanfardino, Dario Iafusco, Assunta S. Rollato, Alessia Piscopo, Stefano Curto, Fortunato Lombardo, Bruno Bombaci, Silvia Sordelli, Chiara Mameli, Maddalena Macedoni, Andrea Rigamonti, Riccardo Bonfanti, Giulio Frontino, Barbara Predieri, Patrizia Bruzzi, Enza Mozzillo, Francesco Rosanio, Adriana Franzese, Gavina Piredda, Francesca Cardella, Brunella Iovane, Valeria Calcaterra, Maria Giulia Berioli, Anna Lasagni, Valentina Pampanini, Patrizia Ippolita Patera, Riccardo Schiaffini, Irene Rutigliano, Gianfranco Meloni, Luisa De Sanctis, Davide Tinti, Michela Trada, Lucia Paola Guerraggio, Roberto Franceschi, Vittoria Cauvin, Gianluca Tornese, Francesca Franco, Gianluca Musolino, Giulio Maltoni, Valentina Talarico, Antonio Iannilli, Lorenzo Lenzi, Maria Cristina Matteoli, Erica Pozzi, Carlo Moretti, Stefano Zucchini, Ivana Rabbone, Rosaria Gesuita

**Affiliations:** ^1^Department of Women’s and Children’s Health, Azienda Ospedaliero-Universitaria, Ospedali Riuniti di Ancona, “G. Salesi Hospital” , Ancona, Italy; ^2^Pediatric Diabetes, Endocrinology and Nutrition, Pediatric Unit, ASST Cremona, Ospedale Maggiore, Cremona, Italy; ^3^Pediatric Unit, Beauregard Hospital, Aosta, Italy; ^4^Metabolic Disease and Genetics Disorders Unit, Giovanni XXIII Children’s Hospital, Bari, Italy; ^5^Maternal and Child Health Department, Pediatric Unit - San Bortolo Hospital, Vicenza, Italy; ^6^Pediatric Clinic, Children’s Hospital, ASST Spedali Civili Brescia, Brescia, Italy; ^7^Unit of Pediatrics, Perrino Hospital, Brindisi, Italy; ^8^Pediatric Diabetology Unit, Pediatric and Microcytemia Department, AO Brotzu, Cagliari, Italy; ^9^Pediatric Endocrinology Unit, Sant”Anna e San Sebastiano Hospital, Caserta, Italy; ^10^Unit of Pediatrics, Spoke Hospital, Castrovillari, Cozensa, Italy; ^11^Department of Pediatrics “Pugliese-Ciaccio” Hospital, Catanzaro, Italy; ^12^UOS Pediatric Diabetology ASL Viterbo, Lazio, Italy; ^13^Unit of Paediatrics, “ M.Bufalini” Hospital Cesena (FC), Unit of Paediatrics, “ S.M. Croci” Hospital Ravenna (RA), AUSL della Romagna Ravenna, Emilia-Romagna, Italy; ^14^Pediatrics Unit, Spoke Hospital Cetraro, Calabria, Italy; ^15^Department of Pediatrics, University of Chieti, Chieti, Italy; ^16^Department of Pediatrics, ASST Lariana, Sant’Anna Hospital, Como, Italy; ^17^UASP Cosenza, Cosenza, Italy; ^18^Pediatric and Pediatric Emergency unit, Children Hospital, ASO SS Antonio Biagio e Cesare Arrigo, Alessandria, Italy; ^19^Unit of Pediatrics ASO S Croce e Carle, Cuneo, Italy; ^20^Diabetology and Endocrinology Unit, Meyer University Children’s Hospital, Florence, Italy; ^21^Unit of Pediatrics, “D. Camberlingo” Hospital, Francavilla Fontana, Brindisi, Italy; ^22^Unit of Pediatrics, “Sacro Cuore di Gesù” Hospital Gallipoli (LE), Gallipoli, Italy; ^23^Pediatric Clinic, IRCCS Giannina Gaslini; Department of Neuroscience Rehabilitation Ophtalmology Genetics, Maternal and Child Health, University of Genoa, Genova, Italy; ^24^Department of Surgery, Dentistry, Pediatrics and Gynecology, Section of Pediatric Diabetes and Metabolism, University and Azienda Ospedaliera Universitaria Integrata of Verona, Verona, Italy; ^25^Department of Woman, Child and General and Specialistic Surgery, Regional Center of Pediatric Diabetes, University of Campania “L. Vanvitelli”, Naples, Italy; ^26^Department of Human Pathology of Adulthood and Childhood G. Barresi, University of Messina, Messina, Italy; ^27^Department of Pediatrics, ASST Carlo Poma, Mantova, Italy; ^28^Department of Pediatrics, V. Buzzi Children's Hospital. Univerisity of Milan, Milano, Lombardia, Italy; ^29^Diabetes Research Institute, IRCCS San Raffaele Hospital, Milan, Italy; ^30^Department of Medical and Surgical Sciences of the Mother, Children and Adults, Pediatric Unit, University of Modena and Reggio Emilia, Modena, Italy; ^31^Department of Translational Medical Science, Section of Pediatrics, Università degli Studi di Napoli Federico II, Naples, Italy; ^32^Unit of Paediatrics, “Giovanni Paolo II“ Hospital, ASSL Olbia, Olbia, Italy; ^33^Department of Pediatrics, Regional Center of Pediatric Diabetes, Children Hospital G. Di Cristina, Palermo, Italy; ^34^Parma University Hospital Department of Mother and Child Pediatric, Parma, Italy; ^35^Department of Internal Medicine and Therapeutics, University of Pavia and “Vittore Buzzi” Chidren’s Hospital, Milano, Italy; ^36^Pediatric Diabetology Department, Azienda Ospedaliera di Perugia, Perugia, Umbria, Italy; ^37^Pediatric Unit, Department of Obstetrics, Gynaecology and Paediatrics, Azienda AUSL-IRCCS Reggio Emilia, Italy; ^38^Pediatric Diabetology Department, Bambino Gesu Pediatric Hospital Roma, Lazio, Italy; ^39^Casa Sollievo della Sofferenza” Research Institut, San Giovanni Rotondo, Puglia, Italy; ^40^Department of Medical Surgical and Experimental Sciences, University of Sassari, Sassari, Italy; ^41^Center of Pediatric Diabetology - A.O.U. Città della Salute e della Scienza di Torino, Torino, Italy; ^42^Centre of Paediatric Diabetology, Paediatric Unit, "Filippo Del Ponte" Children Hospital, ASST Sette Laghi, Varese, Italy; ^43^Department of Pediatrics, S.Chiara Hospital of Trento, Trento, Trentino-Alto Adige, Italy; ^44^Institute for Maternal and Child health IRCCS “Burlo Garofolo”, Trieste, Italy; ^45^Pediatric Department, ASUFC Hospital of Udine, Friuli-Venezia Giulia, Italy; ^46^Centre of Paediatric Diabetology, Paediatric Unit, “Filippo Del Ponte” Children Hospital, ASST Sette Laghi, Varese, Italy; ^47^Pediatric Endocrine Unit, IRCCS Azienda Ospedaliero-Universitaria di Bologna, Bologna, Italy; ^48^Division of Pediatrics, Department of Health Sciences, University of Piemonte Orientale, Novara, Italy; ^49^UOSD Pediatric Diabetology and Metabolism Unit, Children and Women Health Department, AOU Padova, Padova, Italy; ^50^Center of Epidemiology, Biostatistics and Medical Information Technology, Polytechnic University of Marche, Ancona, Italy

**Keywords:** DKA, COVID - 19, type 1 diabetes, socioeconomic status, diabetes onset

## Abstract

**Aim/Hypothesis:**

To compare the frequency of diabetic ketoacidosis (DKA) at diagnosis of type 1 diabetes in Italy during the COVID-19 pandemic in 2020 with the frequency of DKA during 2017-2019.

**Methods:**

Forty-seven pediatric diabetes centers caring for >90% of young people with diabetes in Italy recruited 4,237 newly diagnosed children with type 1 diabetes between 2017 and 2020 in a longitudinal study. Four subperiods in 2020 were defined based on government-imposed containment measures for COVID-19, and the frequencies of DKA and severe DKA compared with the same periods in 2017-2019.

**Results:**

Overall, the frequency of DKA increased from 35.7% (95%CI, 33.5-36.9) in 2017-2019 to 39.6% (95%CI, 36.7-42.4) in 2020 (p=0.008), while the frequency of severe DKA increased from 10.4% in 2017-2019 (95%CI, 9.4-11.5) to 14.2% in 2020 (95%CI, 12.3-16.4, p<0.001). DKA and severe DKA increased during the early pandemic period by 10.4% (p=0.004) and 8% (p=0.002), respectively, and the increase continued throughout 2020. Immigrant background increased and high household income decreased the probability of presenting with DKA (OR: 1.55; 95%CI, 1.24-1.94; p<0.001 and OR: 0.60; 95 CI, 0.41-0.88; p=0.010, respectively).

**Conclusions/Interpretation:**

There was an increase in the frequency of DKA and severe DKA in children newly diagnosed with type 1 diabetes during the COVID-19 pandemic in 2020, with no apparent association with the severity of COVID-19 infection severity or containment measures. There has been a silent outbreak of DKA in children during the pandemic, and preventive action is required to prevent this phenomenon in the event of further generalized lockdowns or future outbreaks.

## Highlights

**What is already known about this subject?**

- The frequency of diabetic ketoacidosis (DKA) at onset of type 1 diabetes was of more than 40% in Italy between 2006 and 2016.
- Early reports suggested an increase of DKA frequency at diagnosis of type 1 diabetes in children.

**What is the key question?**

- Is the frequency of DKA at diagnosis of type 1 diabetes in Italy during the COVID-19 pandemic in 2020 higher than the frequency of DKA during 2017-2019?

**What are the new findings?**

- There was a worrying increase in DKA and severe DKA in children newly diagnosed with type 1 diabetes during the COVID-19 pandemic in 2020 compared with the previous three years.
- There has been no apparent association between the increase in DKA and the containment measures implemented by the Italian government to limit the spread of COVID-19.
- Immigrant background increased and high household income decreased the probability of presenting with DKA.

**How might this impact on clinical practice in the foreseeable future?**

- Strong preventive action is needed to limit this phenomenon in the event of further generalized lockdowns or future outbreaks. Health services should implement awareness campaigns and population screening for type 1 diabetes that have been shown to prevent DKA at diagnosis of type 1 diabetes.

## Introduction

Italy witnessed a very high frequency of diabetic ketoacidosis (DKA) at onset of type 1 diabetes between 2006 and 2016 (1). However, thanks to awareness campaigns such as those promoted by the Italian Society of Pediatric Endocrinology and Diabetology (ISPED), these figures slowly but significant decreased and indeed became more evident between 2014 and 2016 (2). The COVID-19 pandemic has prompted rapid changes in the organization of healthcare systems and public behavior, and there were early reports of a large increase in the frequency of DKA at the time of diagnosis of type 1 diabetes during the pandemic (3–10); indeed, a German study reported that the observed versus predictive frequency of DKA was only higher in the early phase of the pandemic in 2020 (11).

In 2020, Italy suffered the highest total number of deaths since the Second World War, with a 15.6% excess in deaths in 2020 compared with the 2015-2019 period. Although there were fewer reported deaths in January and February 2020, March to December 2020 saw a 21% increase in mortality, with two peaks between March and April and October and December during the two waves of the pandemic (12).

With the aim of controlling the spread of the SARS-CoV-2 virus, the Italian government issued several legislative decrees to limit the movement of people and ensure physical distancing together with economic and social policies to support health and employment (13). Containment measures have alternated between extreme and partial based on the impact of COVID-19 on the population. Extreme measures included limiting individual travel, closing schools and restaurants, banning gatherings, banning interregional, national, and international travel, cancelling public events, and shutting down workplaces, while partial measures included nationally controlled individual driving permits, opening takeaway restaurants, authorized outdoor public events, and opening workplaces. It is currently unclear whether these COVID disease severity and containment measures are related to the frequency of DKA at diabetes diagnosis. Therefore, the purpose of this study was to investigate whether the frequency of DKA at diagnosis of type 1 diabetes changed during the COVID-19 pandemic and was associated with COVID-19 disease severity or the measures taken by government to curb its spread.

## Research Design and Methods

### Study Design and Data Sources

We analyzed data from the ISPED Network for DKA Study and Prevention (2). The Network was established in 2014 following observation of a very high frequency of DKA in Italy (14) with the aim of design studies to continuously analyze DKA frequency and discuss prevention measures. It includes all the Italian centers for childhood and adolescent diabetes, which prospectively record the data of all children at the time of diagnosis of type 1 diabetes according to a procedure agreed in 1997 (15). The Network has annual meetings with local collaborators to discuss and agree on methods and measures for the control and prevention of this harmful complication.

In Italy, pediatric diabetic centers care for 100% of children and adolescents under 15 years with diabetes, over 80% between the 15 and 18 years, and <10% over those aged over 18.

Locally collected pseudonymized longitudinal data are transmitted annually to the coordinating center in Ancona for quality control, plausibility checking, and analysis. Inconsistent data are reported back to participating centers for validation and/or correction. Since 2014, centers have transferred data on clinical, socio-economic, and immigration background to the Network, and since 2017 data on β-cell autoantibody test results from children and adolescents with newly diagnosed type 1 diabetes. Autoantibody measurements are used to confirm patient has type 1 diabetes.

Here we included children and adolescents aged <18 years from 47 diabetes centers in Italy newly diagnosed with type 1 diabetes between 1 January 2017 and 31 December 2020. The diagnosis was confirmed according to the presence of β-cell autoantibodies or the absence of *MODY* mutations and clinical criteria for the diagnosis of type 2 diabetes. DKA was defined as venous pH <7.3 or serum bicarbonate <15 mmol/L or a documented clinical diagnosis of DKA (yes/no) according to the treating physician. Severe DKA was defined as venous pH <7.1 or bicarbonate <5 mmol/L.

**Figure 1:**
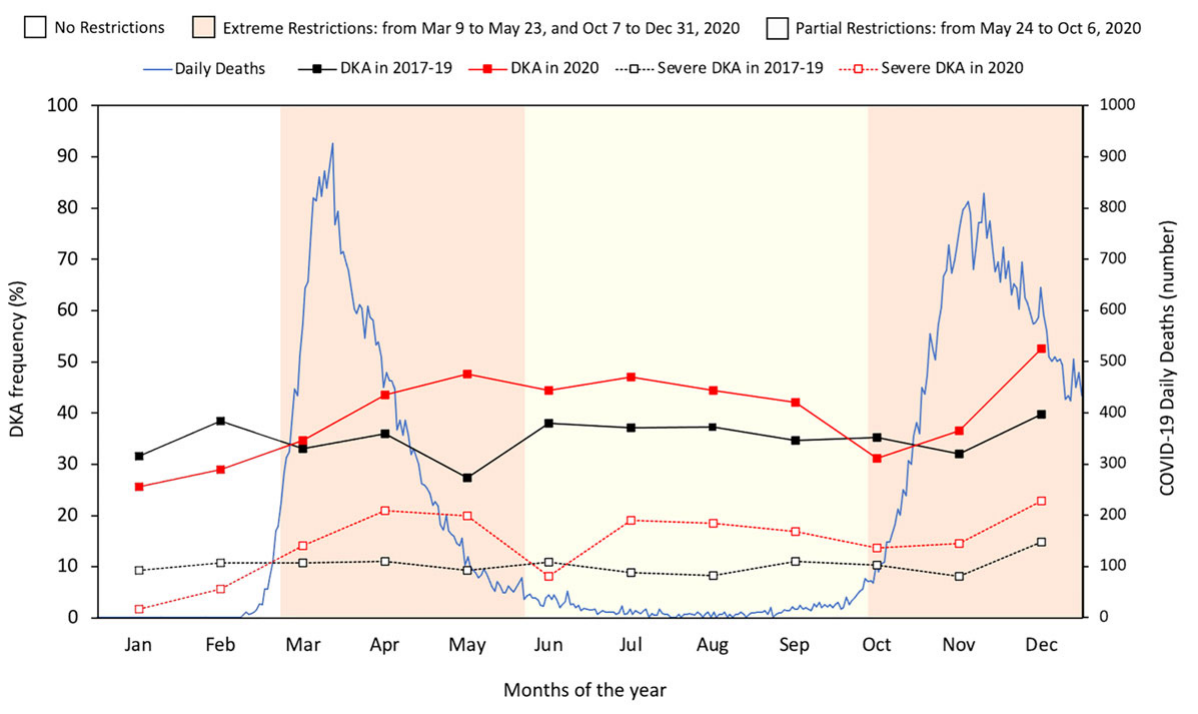
Number of daily deaths from the COVID-19 pandemic in Italy in 2020, and monthly frequency of DKA and severe DKA based on government restriction periods in 2020 compared with the three-year period 2017-2019.

There were two epidemic waves of COVID-19 of different intensity in Italy in 2020: the first between March and April and the second between October and December. The containment measures were significantly changed between March and December 2020 based on the daily number of infections, number of hospital admissions, number of intensive care unit (ICU) admissions, number of deaths, and daily positivity rate. We therefore identified four periods in 2020 that differed in terms of containment measures but with clearly definable characteristics (**Figure 1**): (i) the pre-pandemic period included January and February and ended on March 9, when (ii) the second period started during which the number of daily deaths in the general population increased dramatically and the government imposed extreme measures to contain the pandemic; (ii) the third period ran from May 24 to October 7, during which government-imposed restrictions eased following a sharp reduction in the number of daily deaths; and (iv) the fourth period between October and December was characterized by a rapid increase in daily deaths and the resumption of extreme containment measures.

The frequencies of DKA and severe DKA observed in the four periods of 2020 were compared with the mean frequency observed in the same periods of the calendar year in 2017-2019. Data quality control was performed as previously described (14).

### Variables

Demographic data included age at type 1 diabetes diagnosis (0.5-4; 5-9; 10-14; and 15-18 years), sex, immigration background (defined as the patient and/or one of the parents born outside Italy), and family history of diabetes (defined as at least one parent with type 1 diabetes). Socioeconomic variables included household income (<15.000, 15.000-25.999, 26.000-54.999, 55.000-75.000, >75.000 euros), number of family members, home ownership, and parents’ ages, occupations, and educational level. Clinical data included HbA1c (% and mmol/mol), presence of DKA and severe DKA, and presence of at least one β-cell autoantibody (islet cell antibody, anti-GAD, anti-IA2, IAA, or anti-ZnT8).

### Statistical Analysis

Descriptive statistics were used to characterize children and adolescents at type 1 diabetes diagnosis based on year of disease onset (before COVID-19 pandemic, 2017-2019, and during pandemic in 2020). Household income and parents’ educational level were dichotomized into ≥26,000 euros (high household income) and high school diploma or degree (high educational level), respectively. Variables were summarized using absolute and percentage frequencies for the categorical variables and the means and standard deviations (SD) for the quantitative variables; comparisons between the 2017-19 and 2020 were made using the chi-squared test and Student’s *t*-test for independent samples for categorical and continuous variables, respectively.

Point estimates and 95% confidence intervals (95%CIs) for the frequency of DKA at diabetes onset before and during the COVID-19 pandemic were calculated in each of the four subperiods in 2020 (January-February, March-May, June-September, October-December) using the binomial distributions. Comparisons between the DKA frequencies were performed using the z-test for two proportions.

Logistic regression analysis was used to evaluate the frequency of DKA at diagnosis of type 1 diabetes according to 2020 subperiods compared with the frequency observed in the same months of the preceding three years 2017-2019. Gender, age group at diagnosis, geographical area of residence at diagnosis, family history of type 1 diabetes, immigration background, and the interaction between the four subperiods and years at diagnosis were included in the multiple logistic regression model as covariates.

A multiple logistic regression model was used to estimate the association between DKA frequency and patients’ socioeconomic characteristics in 2020. The final model was obtained using forward criteria and the likelihood ratio test.

Results are expressed as odds ratios (ORs) and 95%CIs. The Hosmer-Lemeshow test was used to assess the goodness of fit of data to the model. Significance was defined as a p-value <0.05 and confidence intervals were set at 95%. All analyses were performed with R-gnu version 4.0.5.

## Results

Overall, 4237 new cases of type 1 diabetes were diagnosed between 2017 and 2020: 3068 in 2017-2019 and 1169 in 2020. There were no missing values in demographic and diagnosis of diabetes, while completeness of DKA data was 99%, HbA1c 96%, beta/cell autoantibody test 92%, family history of diabetes 80%, immigration background 73% and socioeconomic variables between 45 and 55%.

**Supplemental Table S1** shows the characteristics of the new cases of type 1 diabetes according to period of diagnosis. There were no significant differences between the two periods in terms of geographical distribution, gender, age at diagnosis, immigration background, family history of type 1 diabetes, or negative β-cell autoantibody test result. However, frequencies of DKA and severe DKA were significantly higher in 2020 than in the preceding three-year period. Additionally, pH was significantly lower and HbA1c values at type 1 diabetes diagnosis were significantly higher in 2020 than in 2017-2019.

**Table 1** reports estimates of the frequency of DKA, severe DKA, and β-cell autoantibody negativity before and during the COVID-19 pandemic based on the restrictions imposed in 2020. DKA was significantly more frequent during the first period of extreme and partial restrictions in 2020 (10% and 7%, respectively) compared with the same subperiods before the pandemic. The 4% greater frequency observed during the second period of extreme restrictions in 2020 was significantly different to the same period in 2017-2019. During all periods of extreme and partial restrictions, the frequency of severe DKA was significantly higher in 2020 than in 2017-2019. Of note, the frequencies of DKA and severe DKA were significantly lower in January-February 2020 compared with the same months in the previous three-year period. **Figure 1** illustrates the number of daily deaths due to COVID-19 in Italy in 2020 and the monthly frequency of DKA and severe DKA in 2020 compared with in 2017-2019 based on the subperiods marking imposition of national restrictions in 2020.

**Table 1:** Frequency of DKA, severe DKA, and negative b-cell antibody status (95%CI) before and during the COVID-19 pandemic by periods of restrictions.

	2017-2019		2020		
	DKA Cases	% DKA (95%CI)	DKA Cases	% DKA (95%CI)	p-value
**DKA**	1071	35.7 (33.5-36.9)	460	39.6 (36.7-42.4)	0.008
**Jan-Feb**	216	34.8 (31.1-38.8)	57	27.0 (21.3-33.6)	0.045
**Mar-May**	235	32.1 (28.8-35.7)	104	42.4 (36.2-48.9)	0.004
**Jun-Sep**	291	36.6 (33.2-40.0)	152	44.3 (39.0-49.8)	0.017
**Oct-Dec**	329	35.7 (32.6-38.9)	147	39.7 (34.7-44.9)	0.199
**Severe DKA**	319	10.4 (9.4-11.5)	166	14.2 (12.3-16.4)	<0.001
**Jan-Feb**	62	10 (7.8-12.7)	7	3.3 (1.5-7.0)	0.004
**Mar-May**	76	10.4 (8.3-12.9)	45	18.4 (13.8-23.9)	0.002
**Jun-Sep**	78	9.8 (7.9-12.1)	52	15.2 (11.6-19.5)	0.012
**Oct-Dec**	103	11.2 (9.3-13.4)	62	16.8 (13.2-21)	0.009
**b-cell autoantibody negativity**	353	11.5 (10.4-12.7)	137	11.7 (9.9-13.7)	0.888
**Jan-Feb**	74	11.9 (9.5-14.8)	15	7.1 (4.2-11.7)	0.067
**Mar-May**	87	11.9 (9.7-14.5)	23	9.4 (6.2-13.9)	0.337
**Jun-Sep**	84	10.6 (8.5-12.9)	55	16.0 (12.4-20.4)	0.013
**Oct-Dec**	108	11.7 (9.8-14)	44	11.9 (8.9-15.7)	0.999

Seventy-eight percent of participating centers were able to measure at least four out five b-cell autoantibodies, and all the other centers measured more than one b-cell autoantibody. The frequency of b-cell autoantibody negativity was similar in 2020 and 2017-2019, with a significantly higher value observed in June-September 2020 than in the previous period (**Table 1**).

**Table 2** reports the multiple logistic regression analyzing the factors associated with the frequency of DKA and severe DKA. Females were at higher risk (>20%) of DKA at diagnosis of type 1 diabetes than males, and children in the younger age group were at higher risk of DKA and severe DKA than older children. The probability of presenting with DKA was lower in the north of Italy than in central Italy, children with at least one first degree relative with type 1 diabetes were at lower risk of DKA and severe DKA, while children in families with an immigrant background were at higher risk of DKA and severe DKA. The probability of DKA at diagnosis was 2.31-times greater in the first period of extreme restrictions in 2020 compared with the same months in 2017-2019. Children were at significantly higher risk of severe DKA during both the extreme and partial restriction periods in 2020 than in the same months before the COVID-19 pandemic.

**Table 2:** Factors associated with DKA and severe DKA at type 1 diabetes diagnosis.

	DKA			Severe DKA		
	OR	95%CI	p-value	OR	95%CI	p-value
**Intercept**	0.97	0.71-1.32	0.855	0.12	0.08-0.20	<0.001
**2020 vs 2017-2019**	0.89	0.67-1.18	0.154	0.36	0.12-0.86	0.037
**Mar-May vs Jan-Feb**	1.13	0.86-1.48	0.423	1.22	0.77-1.95	0.392
**Jun-Sep vs Jan-Feb**	1.07	0.82-1.39	0.385	1.36	0.87-2.15	0.183
**Oct-Dec vs Jan-Feb**	0.74	0.49-1.11	0.617	1.37	0.89-2.15	0.156
**F vs M**	1.21	1.03-1.41	0.019	1.21	0.95-1.53	0.117
**5-9 vs 0-4 y**	0.55	0.44-0.68	<0.001	0.49	0.36-0.66	<0.001
**10-14 vs 0-4 y**	0.72	0.58-0.89	0.002	0.60	0.45-0.81	<0.001
**15-18 vs 0-4 y**	0.64	0.45-0.90	0.010	0.31	0.16-0.56	<0.001
**North vs central**	0.69	0.56-0.84	<0.001	1.09	0.80-1.49	0.604
**South vs central**	0.95	0.77-1.18	0.635	1.08	0.77-1.53	0.642
**Family history of type 1 diabetes**	0.40	0.31-0.51	<0.001	0.57	0.37-0.85	0.008
**Immigration background (yes vs no)**	1.55	1.24-1.94	<0.001	1.80	1.32-2.44	<0.001
**Mar-May 2020 vs Mar-May 2017-2019**	2.31	1.34-4.01	0.003	5.61	2.02-18.4	0.002
**Jun-Sep 2020 vs Jun-Sep 2017-2019**	1.68	1.00-2.84	0.052	3.70	1.35-12.0	0.017
**Oct-Dec 2020 vs Oct-Dec 2017-2019**	1.54	0.93-2.57	0.097	4.90	1.84-15.57	0.003

DKA model: Likelihood ratio test c^2^ with 15 degrees of freedom=124.9, p < 0.001; Hosmer-Lemeshow test c ^2^ with 8 degrees of freedom=5.29, p=0.726;

Severe DKA: Likelihood ratio test c ^2^ with 15 degrees of freedom=93.1, p < 0.001; Hosmer-Lemeshow test c ^2^ with 8 degrees of freedom=14.2, p=0.076.

**Table 3** shows associations between the frequency of DKA and factors significant according to logistic regression, i.e., subperiods of restrictions, age at diagnosis, family history of type 1 diabetes, and household income. Due to the missing values the association was analyzed only in 2020. The probability of DKA at diagnosis of type 1 diabetes in the partial and extreme restriction periods was over twice that seen in January and February of 2020. The highest age group at diagnosis, presence of a first degree relative with type 1 diabetes, and family income significantly reduced the probability of DKA at type 1 diabetes diagnosis.

**Table 3:** Factors associated with DKA at type 1 diabetes diagnosis in 2020 during the COVID-19 pandemic.

	DKA		
	OR	95%CI	p-value
**Intercept**	0.56	0.29-1.05	0.076
**Mar-May vs Jan-Feb**	2.65	1.43-5.04	0.002
**Jun-Sep vs Jan-Feb**	2.43	1.33-4.56	0.005
**Oct-Dec vs Jan-Feb**	2.28	1.26-4.22	0.007
**5-9 vs 0-4 y**	0.68	0.39-1.18	0.165
**10-14 vs 0-4 y**	0.92	0.53-1.58	0.753
**15-18 vs 0-4 y**	0.39	0.15-0.97	0.049
**Family history of type 1 diabetes**	0.40	0.21-0.73	0.004
**Household income, high vs low**	0.60	0.41-0.88	0.010

DKA model: Likelihood Ratio test c ^2^ with 8 degrees of freedom=31.8, p < 0.001;

Hosmer-Lemeshow test c ^2^ with 8 degrees of freedom=8.75, p=0.364.

Only 14 of 815 (1.7%) children at type 1 diabetes presentation reported a COVID-19 infection before diabetes, and none required hospitalization. 90% of children were tested by PCR for SARS-CoV-2 at diagnosis of diabetes, and 2.5% were positive. Additionally, in 3.4% of cases, at least one family member reported a COVID-19 infection confirmed before the child was diagnosed with diabetes.

There were two DKA-related deaths during 2017-2019, and none during 2020.

## Discussion

This large national study identifies that there was a worrying increase in DKA and severe DKA in children newly diagnosed with type 1 diabetes during the COVID-19 pandemic in 2020 compared with the previous three years. However, there was no apparent association with COVID-19-related number of deaths or the containment measures imposed by the Italian government.

### Description of the Increase in DKA in Italy and Comparisons With Other Countries

This observed increase is consistent with reports from other studies around the world (3–11). To date, except for the study conducted in Germany (11) and in the Lombardy Region of Italy (3), almost all other studies focused on part of 2020 (4–10), in particular the first wave of the pandemic. Furthermore, many were based on few incident cases of type 1 diabetes (4, 6, 7, 9), so consequently the increase in the frequency of DKA varied enormously.

The observed increase in DKA appears to be independent of the daily number of deaths from COVID-19, as the frequency of DKA remained high even in the third period of the year when the number of COVID-19-related deaths decreased and the frequency of severe DKA increased. Likewise, there was no direct relationship between DKA frequency and the government containment measures imposed by law. In particular, the easing of restrictions between June and September 2020 did not appear to directly reduce DKA frequency. It should be noted that, in line with the decreasing trend previously observed (2), in the first two months of 2020 the frequency of DKA was significantly lower than observed in the same months of the preceding three-year period. This might have been due to the awareness campaigns implemented in several areas of the country.

### Possible Causes of Increased DKA and the Role of Socioeconomic Factors

The most compelling reason explaining the increased frequency of DKA is a delay in diabetes diagnosis, although the reasons for this are not fully understood. Parents’ fear of accessing hospital or health services in general due to the risk of contracting SARS-CoV-2 is likely to have been a major cause of delayed diagnosis. However, delays in performing diagnostic tests or restricted access to health services committed to treating COVID-19 patients may have also contributed.

Any delay in diagnosing life-threatening childhood diseases such as complicated diabetes with severe DKA must be seen as a wake-up call for national health systems. In a recent study (16), there was no evidence of a delay in the diagnosis or increased disease severity for childhood cancers or type 1 diabetes during the first wave of the COVID-19 pandemic in centers in the UK. Therefore, any delays in diagnosing diabetes are heterogeneous according to individual contexts and geography.

If the increased frequency of DKA was solely due to parental fear, it might be expected that months of containment measures, drastic reductions in COVID-19 mortality, reduced pressure on hospitals, and the COVID-19 vaccination rollout would reduce the frequency of DKA. We did not assess a metric of parental fear; however, in the last three months of 2020 and over seven months after the start of the COVID-19 pandemic, the frequency of DKA increased.

This study confirms that DKA and severe DKA at diagnosis of type 1 diabetes are more likely in children with an immigrant background. Analysis of the role of socioeconomic factors showed that people with high household incomes were at lower risk of DKA, suggesting that high socioeconomic status is protective even in times of emergency, such as during the pandemic. We hypothesize that people with higher incomes may favor private health services, assuming that the private system may be easier to access and less exposed to the risk of infection.

### Link Between COVID-19 and Diabetes

There is currently no convincing evidence of a change in the incidence of type 1 diabetes during the COVID-19 pandemic. In theory, COVID-19 could act as an environmental trigger for diabetes in individuals with high genetic risk and pre-existing b-cell autoimmunity. It has also been reported that COVID-19 may have a direct cytotoxic effect on b-cells by binding to the angiotensin converting enzyme 2 (ACE2) receptor or by proteolytic cleavage of the viral spike protein by the serine transmembrane protease 2 (TMPRSS2) (17). However, recent evidence suggests that SARS-CoV-2 is unlikely to directly infect b-cells *in vivo* (18). The increased frequency of DKA cases without b-cell autoantibodies observed in the third period of 2020 requires further in-depth study. It has been hypothesized that the virus could directly damage b-cells without immune system activation. However, in our series, most cases with b-cell-negative autoantibodies had a COVID-19 negative swab at the time of diabetes onset, and COVID-19 autoantibodies were only tested in a small number of patients. A negative PCR test for SARS-CoV-2 does not exclude viral infection, and indeed computed tomography-positive cases of COVID-19 infection in patients with SARS-CoV-2 RNA-negative tests have been described (19). To better understand this result, more high-quality, long-term data need to be collected and analyzed.

### Limitations and Strengths

This study is strengthened of by the participation of most pediatric diabetes centers in Italy, all of which have been involved for some time in the study and management of DKA in newly diagnosed type 1 diabetes. The study is, however, limited by not considering potential confounding factors such as the presence of COVID-19 infection in family members of children newly diagnosed with type 1 diabetes and measurement of parental fear. Furthermore, most centers were only able to measure up to four b-cell autoantibodies.

## Summary

In summary, our results show that there was a silent epidemic of DKA in children in Italy during the pandemic in 2020. This phenomenon must be prevented in the event of any future generalized lockdowns or epidemics. Prevention information campaigns, which were proven to be effective in the pre-pandemic period in reducing this potentially life-threatening acute complication (20), could be a useful tool in the general population when health systems are stressed. Also, telemedicine should be considered when DKA is diagnosed locally and the transfer to specialized centers hindered by different factors. The continuous collection of high-quality, population-based data is essential for a better understanding of the association between COVID-19 and type 1 diabetes and to prevent DKA in children and adolescents.

## Data Availability Statement

The raw data supporting the conclusions of this article will be made available by the authors, without undue reservation.

## Ethics Statement

The studies involving human participants were reviewed and approved by Comitato Etico Regionale delle Marche. Written informed consent to participate in this study was provided by the participants’ legal guardian/next of kin. Written informed consent was obtained from the individual(s), and minor(s)’ legal guardian/next of kin, for the publication of any potentially identifiable images or data included in this article.

## Author Contributions

VCh and RG conceptualized the study, coordinated and supervised data collection, and wrote the draft of the manuscript. RG designed the statistical analysis and analyzed the data. All authors collected data locally, contributed intellectually to the study, and critically reviewed and approved the final version of the manuscript. VCh is the guarantor of this work and, as such, had full access to all the data in the study and takes responsibility for the integrity of the data and the accuracy of the data analysis.

## Funding

This study was partially supported by The Italian Society of Pediatric Endocrinology and Diabetology.

## Conflict of Interest

No author reported any conflict of interest as regards this study. The following conflicts of interest pointed out are referred to a period from January 2020 to the submission of this manuscript. VCh’s institution has received research grants from AstraZeneca, Novonordisk, Eli Lilly, Movi, Dompè, and Menarini, and VCh received honoraria from Eli Lilly, Tandem, and Insulet for participating on speakers’ bureaus and scientific advisory boards. CR, DT, IRa, BPr, BPi, SZ, ST, and AR has received support Eli Lilly. In addition, SZ’s institution has received support from Pfeizer, ST, BPi, and DT have received support from Abbott and Theras. MM and AR have received support from Menarini. BPr and PB received honoraria for participating on speakers’ bureaus and scientific advisory boards for Sandoz. Lastly, RS has received research grants by Sanofi and received honoraria for participating on speakers’ bureaus and scientific advisory boards for Movi.

The remaining authors declare that the research was conducted in the absence of any commercial or financial relationships that could be construed as a potential conflict of interest.

## Publisher’s Note

All claims expressed in this article are solely those of the authors and do not necessarily represent those of their affiliated organizations, or those of the publisher, the editors and the reviewers. Any product that may be evaluated in this article, or claim that may be made by its manufacturer, is not guaranteed or endorsed by the publisher.

## Abbreviations

DKA, Diabetic Ketoacidosis; ISPED, Italian Society of Pediatric Endocrinology and Diabetology; ICU, Intensive Care Unit; SD, Standard Deviations; CIs, Confidence intervals; ACE2, Angiotensin Converting Enzyme 2; TMPRSS2, Serine Transmembrane Protease 2.
