# Corrigendum: The silent epidemic of diabetic ketoacidosis at diagnosis of type 1 diabetes in children and adolescents in italy during the covid-19 pandemic in 2020

**DOI:** 10.3389/fendo.2022.977211

**Published:** 2022-08-04

**Authors:** Valentino Cherubini, Monica Marino, Andrea E. Scaramuzza, Valentina Tiberi, Adriana Bobbio, Maurizio Delvecchio, Elvira Piccinno, Federica Ortolani, Stefania Innaurato, Barbara Felappi, Francesco Gallo, Carlo Ripoli, Maria Rossella Ricciardi, Filomena Pascarella, Filomena A. Stamati, Felice Citriniti, Claudia Arnaldi, Sara Monti, Vanna Graziani, Fiorella De Berardinis, Cosimo Giannini, Francesco Chiarelli, Maria Zampolli, Rosaria De Marco, Giulia Patrizia Bracciolini, Caterina Grosso, Valeria De Donno, Barbara Piccini, Sonia Toni, Susanna Coccioli, Giuliana Cardinale, Marta Bassi, Nicola Minuto, Giuseppe D’Annunzio, Claudio Maffeis, Marco Marigliano, Angela Zanfardino, Dario Iafusco, Assunta S. Rollato, Alessia Piscopo, Stefano Curto, Fortunato Lombardo, Bruno Bombaci, Silvia Sordelli, Chiara Mameli, Maddalena Macedoni, Andrea Rigamonti, Riccardo Bonfanti, Giulio Frontino, Barbara Predieri, Patrizia Bruzzi, Enza Mozzillo, Francesco Rosanio, Adriana Franzese, Gavina Piredda, Francesca Cardella, Brunella Iovane, Valeria Calcaterra, Maria Giulia Berioli, Anna Lasagni, Valentina Pampanini, Patrizia Ippolita Patera, Riccardo Schiaffini, Irene Rutigliano, Gianfranco Meloni, Luisa De Sanctis, Davide Tinti, Michela Trada, Lucia Paola Guerraggio, Roberto Franceschi, Vittoria Cauvin, Gianluca Tornese, Francesca Franco, Gianluca Musolino, Giulio Maltoni, Valentina Talarico, Antonio Iannilli, Lorenzo Lenzi, Maria Cristina Matteoli, Erica Pozzi, Carlo Moretti, Stefano Zucchini, Ivana Rabbone, Rosaria Gesuita

**Affiliations:** ^1^Department of Women’s and Children’s Health, Azienda Ospedaliero-Universitaria, Ospedali Riuniti di Ancona, “G. Salesi Hospital”, Ancona, Italy; ^2^Pediatric Diabetes, Endocrinology and Nutrition, Pediatric Unit, ASST Cremona, Ospedale Maggiore, Cremona, Italy; ^3^Pediatric Unit, Beauregard Hospital, Aosta, Italy; ^4^Metabolic Disease and Genetics Disorders Unit, Giovanni XXIII Children’s Hospital, Bari, Italy; ^5^Maternal and Child Health Department, Pediatric Unit - San Bortolo Hospital, Vicenza, Italy; ^6^Pediatric Clinic, Children’s Hospital, ASST Spedali Civili Brescia, Brescia, Italy; ^7^Unit of Pediatrics, Perrino Hospital, Brindisi, Italy; ^8^Pediatric Diabetology Unit, Pediatric and Microcytemia Department, AO Brotzu, Cagliari, Italy; ^9^Pediatric Endocrinology Unit, Sant”Anna e San Sebastiano Hospital, Caserta, Italy; ^10^Unit of Pediatrics, Spoke Hospital, Castrovillari, Cozensa, Italy; ^11^Department of Pediatrics “Pugliese-Ciaccio” Hospital, Catanzaro, Italy; 12 UOS Pediatric Diabetology ASL Viterbo, Lazio, Italy; ^13^Unit of Paediatrics, “ M.Bufalini” Hospital Cesena (FC), Unit of Paediatrics, “ S.M. Croci” Hospital Ravenna (RA), AUSL della Romagna Ravenna, Emilia-Romagna, Italy; ^14^Pediatrics Unit, Spoke Hospital Cetraro, Calabria, Italy; ^15^Department of Pediatrics, University of Chieti, Chieti, Italy; ^16^Department of Pediatrics, ASST Lariana, Sant’Anna Hospital, Como, Italy; ^17^UASP Cosenza, Cosenza, Italy; ^18^Pediatric and Pediatric Emergency unit, Children Hospital, ASO SS Antonio Biagio e Cesare Arrigo, Alessandria, Italy; ^19^Unit of Pediatrics ASO S Croce e Carle, Cuneo, Italy; ^20^Diabetology and Endocrinology Unit, Meyer University Children’s Hospital, Florence, Italy; ^21^Unit of Pediatrics, “D. Camberlingo” Hospital, Francavilla Fontana, Brindisi, Italy; ^22^Unit of Pediatrics, “Sacro Cuore di Gesu” Hospital Gallipoli (LE), Gallipoli, Italy; ^23^Pediatric Clinic, IRCCS Giannina Gaslini; Department of Neuroscience Rehabilitation Ophtalmology Genetics, Maternal and Child Health, University of Genoa, Genova, Italy; ^24^Department of Surgery, Dentistry, Pediatrics and Gynecology, Section of Pediatric Diabetes and Metabolism, University and Azienda Ospedaliera Universitaria Integrata of Verona, Verona, Italy; ^25^Department of Woman, Child and General and Specialistic Surgery, Regional Center of Pediatric Diabetes, University of Campania “L. Vanvitelli”, Naples, Italy; ^26^Department of Human Pathology of Adulthood and Childhood G. Barresi, University of Messina, Messina, Italy; ^27^Department of Pediatrics, ASST Carlo Poma, Mantova, Italy; ^28^Department of Pediatrics, V. Buzzi Children’s Hospital. Univerisity of Milan, Milano, Lombardia, Italy; ^29^Diabetes Research Institute, IRCCS San Raffaele Hospital, Milan, Italy; ^30^Department of Medical and Surgical Sciences of the Mother, Children and Adults, Pediatric Unit, University of Modena and Reggio Emilia, Modena, Italy; ^31^Department of Translational Medical Science, Section of Pediatrics, Universita degli Studi di Napoli Federico II, Naples, Italy; ^32^Unit of Paediatrics, “Giovanni Paolo II” Hospital, ASSL Olbia, Olbia, Italy; ^33^Department of Pediatrics, Regional Center of Pediatric Diabetes, Children Hospital G. Di Cristina, Palermo, Italy; ^34^Parma University Hospital Department of Mother and Child Pediatric, Parma, Italy; ^35^Department of Internal Medicine and Therapeutics, University of Pavia and “Vittore Buzzi” Chidren’s Hospital, Milano, Italy; ^36^Pediatric Diabetology Department, Azienda Ospedaliera di Perugia, Perugia, Umbria, Italy; ^37^Pediatric Unit, Department of Obstetrics, Gynaecology and Paediatrics, Azienda AUSL-IRCCS Reggio Emilia, Italy; ^38^Pediatric Diabetology Department, Bambino Gesu Pediatric Hospital Roma, Lazio, Italy; ^39^Casa Sollievo della Sofferenza” Research Institut, San Giovanni Rotondo, Puglia, Italy; ^40^Department of Medical Surgical and Experimental Sciences, University of Sassari, Sassari, Italy; ^41^Center of Pediatric Diabetology - A.O.U. Citta della Salute e della Scienza di Torino, Torino, Italy; ^42^Centre of Paediatric Diabetology, Paediatric Unit, “Filippo Del Ponte” Children Hospital, ASST Sette Laghi, Varese, Italy; ^43^Department of Pediatrics, S.Chiara Hospital of Trento, Trento, Trentino-Alto Adige, Italy; ^44^Institute for Maternal and Child health IRCCS “Burlo Garofolo”, Trieste, Italy; ^45^Pediatric Department, ASUFC Hospital of Udine, Friuli-Venezia Giulia, Italy; ^46^Centre of Paediatric Diabetology, Paediatric Unit, “Filippo Del Ponte” Children Hospital, ASST Sette Laghi, Varese, Italy; ^47^Pediatric Endocrine Unit, IRCCS Azienda Ospedaliero-Universitaria di Bologna, Bologna, Italy; ^48^Division of Pediatrics, Department of Health Sciences, University of Piemonte Orientale, Novara, Italy; ^49^UOSD Pediatric Diabetology and Metabolism Unit, Children and Women Health Department, AOU Padova, Padova, Italy; ^50^Center of Epidemiology, Biostatistics and Medical Information Technology, Polytechnic University of Marche, Ancona, Italy

**Keywords:** DKA, COVID - 19, type 1 diabetes, socioeconomic status, diabetes onset

In the published article, there was an error in affiliation(s) 29. Instead of “Departement of Pediatrics, Diabetes Research Institute, IRCCS San Raffaele, Milano, Italy”, it should be “Diabetes Research Institute, IRCCS San Raffaele Hospital, Milan, Italy”.

The authors apologize for this error and state that this does not change the scientific conclusions of the article in any way. The original article has been updated.

## Publisher’s note

All claims expressed in this article are solely those of the authors and do not necessarily represent those of their affiliated organizations, or those of the publisher, the editors and the reviewers. Any product that may be evaluated in this article, or claim that may be made by its manufacturer, is not guaranteed or endorsed by the publisher.

