# Supplementary material for: The Silent Epidemic of Diabetic Ketoacidosis at Diagnosis of Type 1 Diabetes in Children and Adolescents in Italy During the COVID-19 Pandemic in 2020
Source: Front Endocrinol (Lausanne). 2022 Jun 17;13:878634. doi: 10.3389/fendo.2022.878634 (PMC9247264; doi:10.3389/fendo.2022.878634)
Supplement: Supplementary file 1 [file Table_1.docx]

| **Variables** | **2017-2019** | **2020** | **p** |
| --- | --- | --- | --- |
| **Area of residence at T1D diagnosis, n (%)** | n=3068 | n=1169 |  |
| **North** | 1346 (43.9) | 520 (44.5) | 0.832 |
| **Centre** | 621 (20.2) | 227 (19.4) |  |
| **South** | 1101 (35.9) | 422 (36.1) |  |
| **Gender, n (%)** | n=3068 | n=1169 |  |
| **Males** | 1671 (54.6) | 658 (56.3) | 0.314 |
| **Age at onset, n (%)** | n=3068 | n=1169 |  |
| **0-4 y** | 588 (19.2) | 234 (20) | 0.934 |
| **5-9 y** | 1091 (35.6) | 411 (35.2) |  |
| **10-14 y** | 1145 (37.3) | 430 (36.8) |  |
| **15-18 y** | 244 (8.0) | 94 (8.0) |  |
| **Family history of type 1 diabetes, n (%)** | n=2449 | n=959 |  |
| **Yes** | 323 (13.2) | 130 (13.6) | 0.820 |
| **b-cell autoantibody test, n (%)** | n=2816 | n=1102 |  |
| **Negative** | 353 (12.5) | 137 (12.4) | 0.973 |
| **DKA, Y, n (%)** | n=3045 | n=1163 |  |
| **Yes** | 1071 (35.7) | 460 (39.6) | 0.009 |
| **Severe DKA, n (%)** | n=2928 | n=1131 |  |
| **Yes** | 320 (10.9) | 166 (14.7) | 0.001 |
| **pH at T1D onset** | n=2928 | n=1131 |  |
| **mean (sd)** | 7.29 (0.14) | 7.26 (0.15) | <0.001^#^ |
| **HbA1c at T1D onset** | n=2923 | n=1125 |  |
| **mean (sd)** | 11.2 (2.4) | 11.5 (2.4) | 0.002^#^ |
| **Immigration background, n (%)** | n=2231 | n=867 |  |
| **Yes** | 309 (13.9) | 137 (15.8) | 0.183 |
| **Household income, n (%)** | n=1321 | n=528 |  |
| **High** | 580 (43.9) | 264 (50.0) | 0.020 |
| **Own home, n (%)** | n=1345 | n=544 |  |
| **Yes** | 935 (69.5) | 392 (72.1) | 0.299 |
| **Father age** | n=1977 | n=772 |  |
| **mean (sd)** | 44.3 (7.0) | 45.3 (6.8) | 0.002 |
| **Father educational level, n (%)** | n=1671 | n=646 |  |
| **High** | 880 (52.7) | 375 (58.0) | 0.022 |
| **Mother age** | n=2020 | n=804 |  |
| **mean (sd)** | 40.9 (6.7) | 41.3 (6.9) | 0.125 |
| **Mother educational level, n (%)** | n=1672 | n=644 |  |
| **High** | 1018 (60.9) | 433 (67.2) | 0.005 |

p-values refer to Chis-quare test or ^#^Student-t test for independent samples
Summary statistics are calculated on available data, missing values excluded.
